# A Comparative Evaluation of Bone Density Surrounding Buccally and Palatally Impacted Canines Utilizing Fractal Analysis: A Retrospective Cone-Beam Computed Tomography (CBCT) Study

**DOI:** 10.7759/cureus.82761

**Published:** 2025-04-22

**Authors:** Manish S Agrawal, Sharaniya S Nambiar, Jiwanasha Agrawal, Shraddha S Shetti, Sangamesh Fulari

**Affiliations:** 1 Department of Orthodontics and Dentofacial Orthopedics, Bharati Vidyapeeth (Deemed to be University) Dental College and Hospital, Sangli, IND; 2 Department of Orthodontics, Bharati Vidyapeeth (Deemed to be University) Dental College and Hospital, Sangli, IND

**Keywords:** buccally impacted canine, canine impaction, cbct study, fractal analysis, palatally impacted canine

## Abstract

Introduction: Previous studies have identified environmental and genetic causes of impaction; however, none of these studies have considered bone density as a factor. The study aimed to determine the bone density surrounding buccally and palatally impacted canines and to determine whether bone density is a contributing factor to the higher occurrence of palatal impaction.

Methods: The study sample included 20 pretreatment cone-beam computed tomography (CBCT) scans with either unilateral or bilateral impacted canines. The fractal dimension (FD) was determined using ImageJ software (National Institutes of Health, Bethesda, MD), with a focus on the interproximal region between the first and second premolars, adjacent to the impacted canine. To assess significant differences between the groups, intergroup comparisons were performed using independent samples t-tests and unpaired t-tests.

Results: The mean FD of palatally impacted canines was 1.5563, which was greater than that of buccally impacted canines (1.3983). The overall changes in FD were significant (p = 0.001).

Conclusion: Our analysis of the CBCT images revealed that bone density is higher around canines that are impacted palatally compared to those impacted buccally. Therefore, bone density is one of the local etiologic factors associated with the higher prevalence of palatal impactions.

## Introduction

Maxillary canine impaction is the second most commonly impacted tooth after the third molars. An impacted tooth other than third molars should be aligned for reasons of arch length deficiency, pathological conditions, or for its location that does not allow its alignment. Maxillary canine impactions can be palatal or buccal. The incidence of palatal impaction is higher than buccal impaction [1].

The etiology of impaction can broadly be classified into local or systemic causes. Local causes include arch length deficiency, genetic causes, or increased bone density. Cone-beam computed tomography (CBCT) is one of the methods used for the localization of canines. Advantages of CBCT are three-dimensional images, less image distortion, and low radiation dose. There are only a few studies assessing the bone quality of the maxilla on the impacted and contralateral nonimpacted side. This may be due to Hounsfield units of CBCT, which are not reliable for assessing the bone density [2].

Fractal analysis is a method used in describing complex shapes and structural patterns and is expressed numerically as the fractal dimension (FD). Fractal analysis is the process of information processing, where the data are uploaded in the form of an image [3]. Due to the irregular and random nature of trabecular bone, fractal analysis is a reliable method of assessing bone quality compared to Hounsfield units [1].

There are no previous studies comparing the bone density around buccal and palatal impacted canines. The study aimed to determine the bone density around buccally and palatally impacted canines and to assess whether bone density is an etiologic factor contributing to the increased incidence of palatal impaction.

## Materials and methods

**Figure 1:**
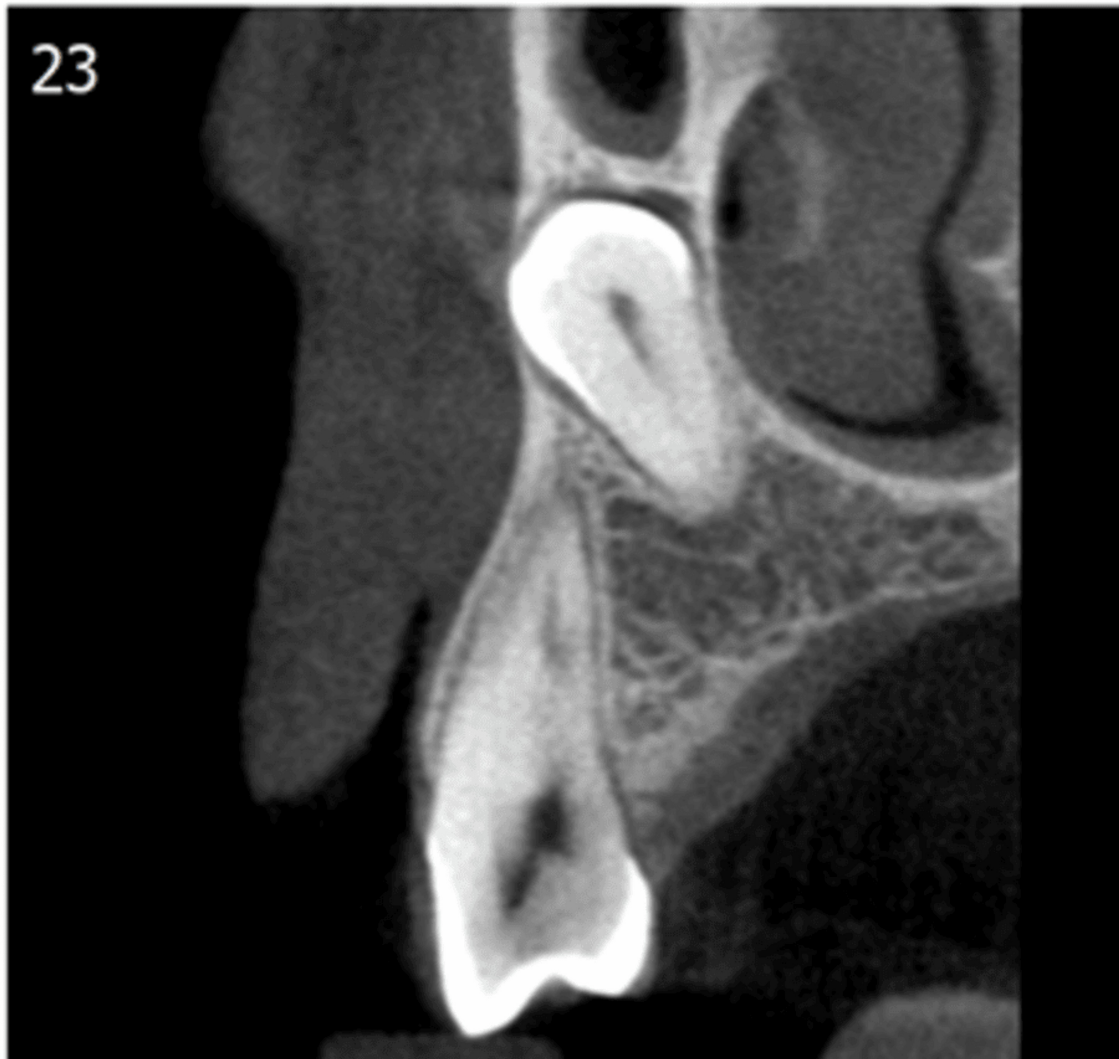
CBCT image (coronal section) showing maxillary impacted canine, which is selected for checking bone density CBCT: cone-beam computed tomography

This retrospective study was done using pretreatment CBCT scans of patients with unilateral or bilateral canine impaction. Twenty CBCT scans (10 buccal and 10 palatal) were selected. Inclusion criteria included CBCT scans with unilateral or bilateral canine impaction, complete eruption of the contralateral canine in unilateral impaction, and no prior history of orthodontic treatment. CBCT scans that included bone pathology, supernumerary teeth, and systemic disease affecting bone health were excluded. The image processing was done using ImageJ software (National Institutes of Health, Bethesda, MD). The area of interest was the interproximal area adjacent to the impacted canine between the first and second premolar because of the availability of trabecular bone (Figure 1).

**Figure 2:**
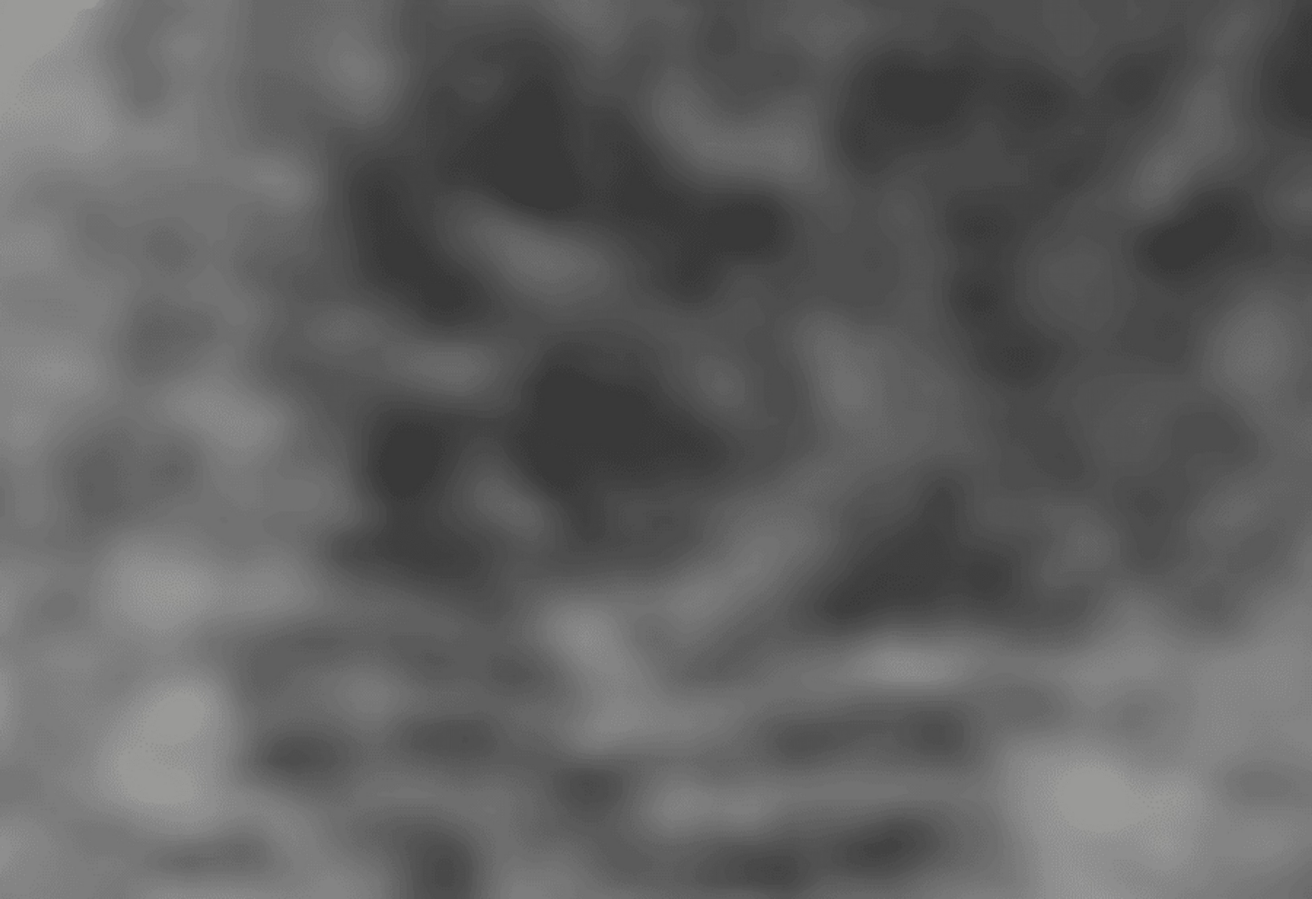
Cropped image of the selected area used for image processing in ImageJ software

CBCT images were saved in JPEG format. The images were cropped to a 64 × 64-pixel region of interest (Figure 2).

**Figure 3:**
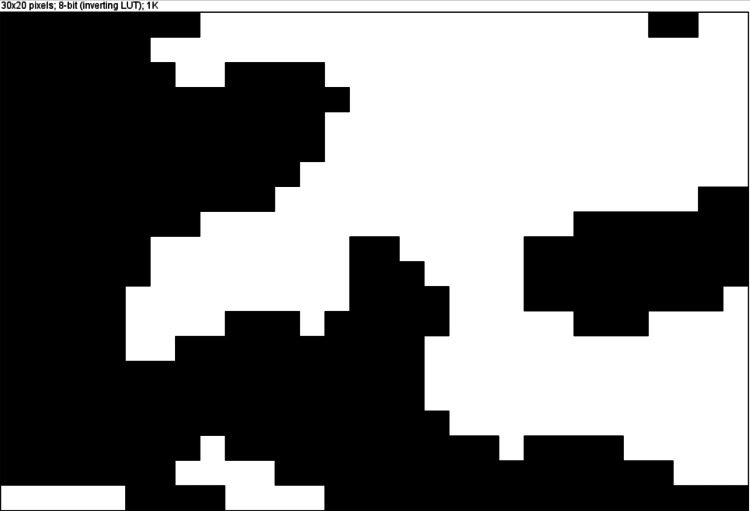
The processed image getting converted to white and black binary image in ImageJ software. The black area denotes trabecular bone and white represents bone marrow

The cropped image was binarized in the ImageJ software. Due to the varying thickness of bone and overlying soft tissue, a Gaussian blur was used to distract from brightness. The processed image gets converted into a black and white binary image (Figure 3).

**Figure 4:**
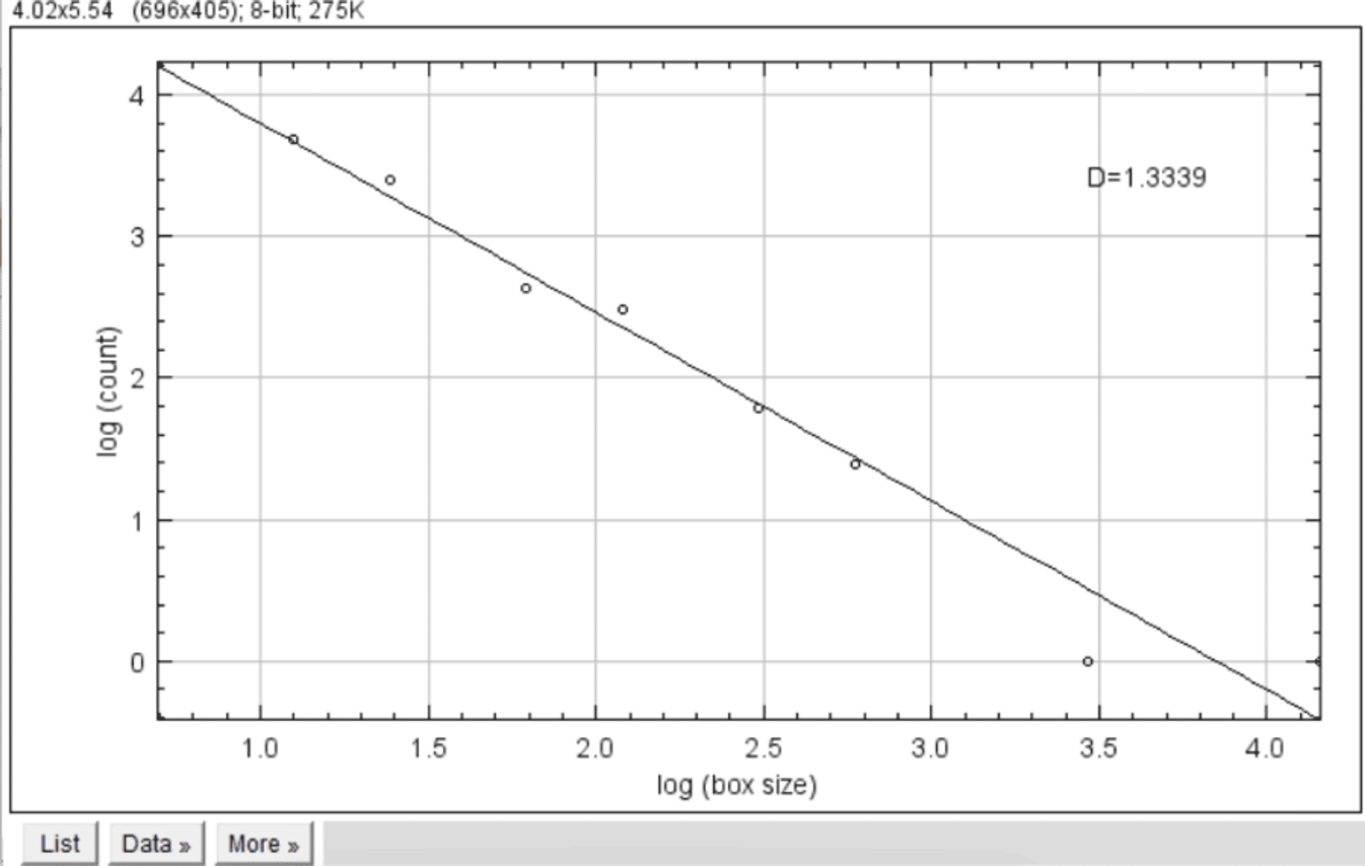
Fractal box count represented as d value

The FD was calculated using the fractal box count (Figure 4). The d value represents the fractal bone density.

## Results

**Figure 5:**
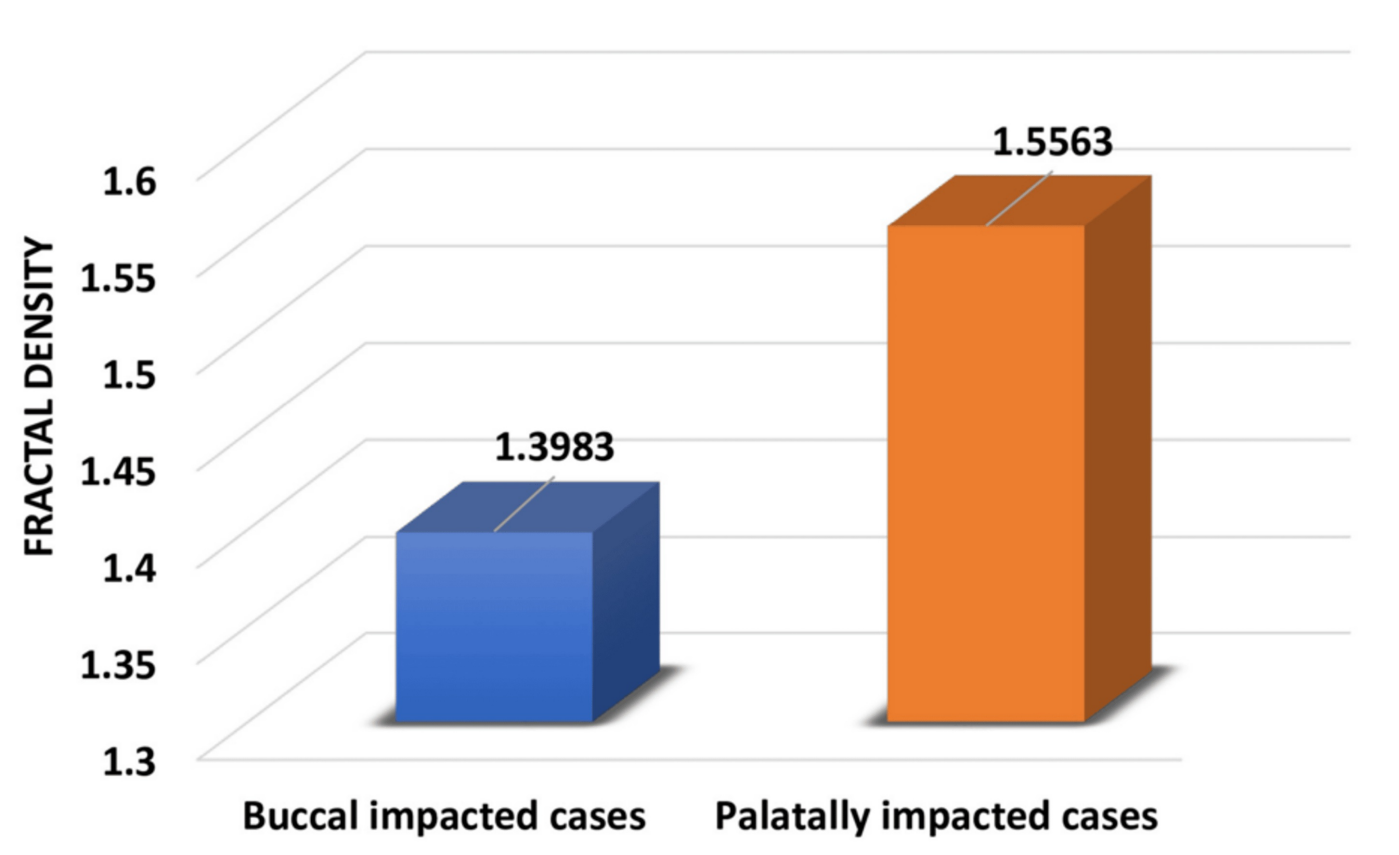
Mean fractal density in buccal and palatally impacted canines

A total of 20 patients were included in the study, out of which 10 were buccally impacted and 10 were palatally impacted cases. The comparison of buccally and palatally impacted canines showed that bone density is increased on the palatal side (Figure 5).

In our study, the intergroup comparison of fractal density between buccally impacted cases and palatally impacted cases was performed using an independent samples t-test. This comparison showed statistically significant differences (p < 0.05) between the two groups (Tables 1, 2).

**Table 1:** Descriptive statistics of fractal density in buccal impacted cases and palatally impacted cases

Groups	n	Minimum	Maximum	Mean	Standard deviation
Buccal impacted cases	10	1.33	1.48	1.3983	0.045
Palatally impacted cases	10	1.39	1.63	1.5563	0.076

**Table 2:** Intergroup comparison of fractal density between buccal impacted cases and palatally impacted cases ^*^Ap value of <0.05 is statistically significant

Parameter	Comparison groups	n	Mean	Mean difference	t	df	p value
Fractal density	Buccal impacted	10	1.3983	-0.158	-5.585	18	0.001^*^
	Palatally impacted	10	1.5563				

## Discussion

This study concluded that the bone density around the palatally impacted canine was greater than that of the buccally impacted canine. Palatally impacted canines have been associated with increased dental arch space, and buccally impacted canines are associated with decreased space for eruption [4-6]. However, there are no previous studies comparing bone density between the palatally and buccally impacted canines.

According to a study conducted by Servais et al., the maxillary alveolar bone area is increased in the impacted side compared with the nonimpacted side [1]. A retrospective study conducted by Arvind et al. concluded that the fractal bone density around impacted canines is greater than that of the nonimpacted side when studied in a Dravidian population. This study also addresses the various uses of fractal density, such as the determination of mid-palatal suture maturation and condylar patterns [7].

CBCT is an emerging technique used for localizing canines. Because Hounsfield was not reliable due to varying amounts of gray scale [2], FD was considered for measuring bone density. Previous studies have cited only genetics [8,9] and the presence of lateral incisors [10] as etiologic factors for impaction; however, no studies have been conducted to consider bone density as a local etiologic factor for the increased incidence of palatal impaction. The rate of tooth movement is inversely proportional to bone density. Thus, knowing the bone density pattern around impacted canines will help in planning any adjunctive acceleratory procedures during disimpaction, thereby reducing treatment time [7].

The study's only limitation was its limited sample size; however, the strict inclusion and exclusion criteria helped maintain the homogeneity of the sample.

## Conclusions

Our analysis of the CBCT scans concluded that bone density around palatally impacted canine is greater as compared to buccally impacted canine. FD analysis in orthodontics is a valuable tool for assessing bone density and trabecular patterns around impacted canines. Thus, bone density is a one of the local etiologic factors for increased incidence of palatal impactions. Thus, knowing the bone density helps in planning a tailored treatment approach for disimpaction and thus helps in improving the biomechanics and treatment outcomes.
